# Arousal Rather than Basic Emotions Influence Long-Term Recognition Memory in Humans

**DOI:** 10.3389/fnbeh.2016.00198

**Published:** 2016-10-21

**Authors:** Artur Marchewka, Marek Wypych, Abnoos Moslehi, Monika Riegel, Jarosław M. Michałowski, Katarzyna Jednoróg

**Affiliations:** ^1^Laboratory of Brain Imaging, Neurobiology Centre, Nencki Institute of Experimental Biology of Polish Academy of SciencesWarsaw, Poland; ^2^Faculty of Psychology, University of WarsawWarsaw, Poland; ^3^Laboratory of Psychophysiology, Department of Neurophysiology, Nencki Institute of Experimental Biology of Polish Academy of SciencesWarsaw, Poland

**Keywords:** long-term memory, basic emotions, affective dimensions, fMRI, affective pictures, Nencki Affective Picture System

## Abstract

Emotion can influence various cognitive processes, however its impact on memory has been traditionally studied over relatively short retention periods and in line with dimensional models of affect. The present study aimed to investigate emotional effects on long-term recognition memory according to a combined framework of affective dimensions and basic emotions. Images selected from the Nencki Affective Picture System were rated on the scale of affective dimensions and basic emotions. After 6 months, subjects took part in a surprise recognition test during an fMRI session. The more negative the pictures the better they were remembered, but also the more false recognitions they provoked. Similar effects were found for the arousal dimension. Recognition success was greater for pictures with lower intensity of happiness and with higher intensity of surprise, sadness, fear, and disgust. Consecutive fMRI analyses showed a significant activation for remembered (recognized) vs. forgotten (not recognized) images in anterior cingulate and bilateral anterior insula as well as in bilateral caudate nuclei and right thalamus. Further, arousal was found to be the only subjective rating significantly modulating brain activation. Higher subjective arousal evoked higher activation associated with memory recognition in the right caudate and the left cingulate gyrus. Notably, no significant modulation was observed for other subjective ratings, including basic emotion intensities. These results emphasize the crucial role of arousal for long-term recognition memory and support the hypothesis that the memorized material, over time, becomes stored in a distributed cortical network including the core salience network and basal ganglia.

## Introduction

Each day we are exposed to a plethora of complex visual scenes and events, yet only a minute portion of these can be successfully retrieved from memory after a long time. Various studies have demonstrated that emotionally arousing incidents and stimuli are much better remembered than those without emotional relevance (see McGaugh, 2004; Holland and Kensinger, 2013; Dolcos and Denkova, 2014 for a review). Importantly, these general findings have been replicated using various methodological approaches and experimental materials, mainly pictures and words (e.g., Bradley et al., 1992; Cahill and McGaugh, 1995; Ochsner, 2000; Kensinger and Corkin, 2003; Dolcos et al., 2004). Recent psychological and neuroscientific studies have also demonstrated that emotion influences not only the efficacy of memory, but various aspects of other cognitive processes, such as perception, attention, or decision-making (see Brosch et al., 2013; Dolcos et al., 2014 for a review). However, recognition memory may be significantly influenced by age-related differences in the processing of emotionally valenced information which have been recently reported (Kalpouzos et al., 2012; Reed et al., 2014; Ziaei and Fischer, 2016 for review).

It is interesting that only several long-term memory experiments have been conducted using a lengthy retention period. In one of the first studies investigating memory after around 1 year, Bradley et al. (1992) discovered a significantly better memory performance for both negative (low-valence), and positive (high-valence) highly arousing stimuli. In a functional resonance imaging (fMRI) study on female subjects after 1-year delay, Dolcos et al. (2005) found a significant improvement of recognition memory for emotional pictures in comparison to neutral ones. Analyses based on the regions of interest (ROI) revealed that successful retrieval of emotional pictures elicited higher activation in the amygdala, enthorinal cortex, and hippocampus. More recently, Weymar et al. (2011) have used behavioral and event-related potential (ERP) measures in passive viewing paradigm with a recognition test conducted 1 week and 1 year after encoding. The results obtained in a group of males showed a sustained enhanced recognition performance for emotionally unpleasant and pleasant pictures as compared to the neutral ones on both retention periods. On the neuronal level, boosted memory for unpleasant and pleasant stimuli after 1 week was related to an enhanced parietal ERP old/new effect. However, after 1 year, these neurophysiological effects were observed only for unpleasant, and not for pleasant, arousing pictures. In an fMRI study conducted on a group of males and females with intentional learning paradigm, valence was found to modulate brain responses after 3 days and 3 months (Harand et al., 2012). This study showed that hippocampal activations can decrease over time and the reorganization of memory traces takes place with additional activation in the ventromedial prefrontal and anterior cingulate cortex.

However, the effect of emotion on long-term recognition memory was only investigated in line with the dimensional models of affect, which describe emotions in a two-dimensional space consisting of valence (pleasantness or unpleasantness) and arousal (level of excitation (Osgood et al., 1957; Russell, 1980). These models are not the only way to conceptualize emotion. Another theoretical framework is Ekman's (1992) theory of basic emotions, according to which at least five different discrete emotion categories universally reflect facial and vocal expression, namely: happiness, sadness, anger, fear, and disgust. These two points of view are not mutually exclusive, having considerable explanatory value (Reisenzein, 1994) and at least two databases of emotionally charged images—International Affective Picture System (IAPS, Libkuman et al., 2007) and Nencki Affective Picture System (NAPS, Marchewka et al, 2014a; Riegel et al., 2016a,b) employ ratings in line with both models. Thus, together dimensional and categorical models of affect should be employed in the studies in order to understand the impact of emotion of cognitive processes (Mikels et al., 2005; Stevenson et al., 2007; Stevenson and James, 2008; Briesemeister et al., 2011).

Some insights into the differential influence of basic emotions on memory and attention can be gained from few existing behavioral and neuroimaging studies. It has been found that stimuli provoking disgust are later recalled better than stimuli provoking fear, and this applied both to words (Charash and McKay, 2002) and images (Croucher et al., 2011; Chapman et al., 2012). Moreover, disgust-evoking images, but not fear-evoking ones, keep hold of our attention for longer (Van Hooff et al., 2013). At the neuronal level, using fMRI and the directed forgetting paradigm, it was revealed that correctly recognized disgusting stimuli evoked the highest activity in the left amygdala compared to all other categories. This structure was also more activated for remembered vs. forgotten stimuli, but only in case of disgust or fear eliciting pictures (Marchewka et al., 2016).

To the best of our knowledge, there has been not a single study investigating the influence of emotion on long-term recognition memory using both dimensional and categorical models of affect.

## Methods

### Material

Overall, 510 images from the NAPS, (Marchewka et al., 2014a) were selected and divided into three subsets, matched in terms of valence and arousal. Each subset consisted of 170 images that proportionally covered the dimensional affective space across various content categories, namely animals, faces, landscapes, objects, and people. A full list of images with the accompanying normative ratings can be found on the NAPS website (http://naps.nencki.gov.pl/).

### Participants and procedure

**Figure 1:**
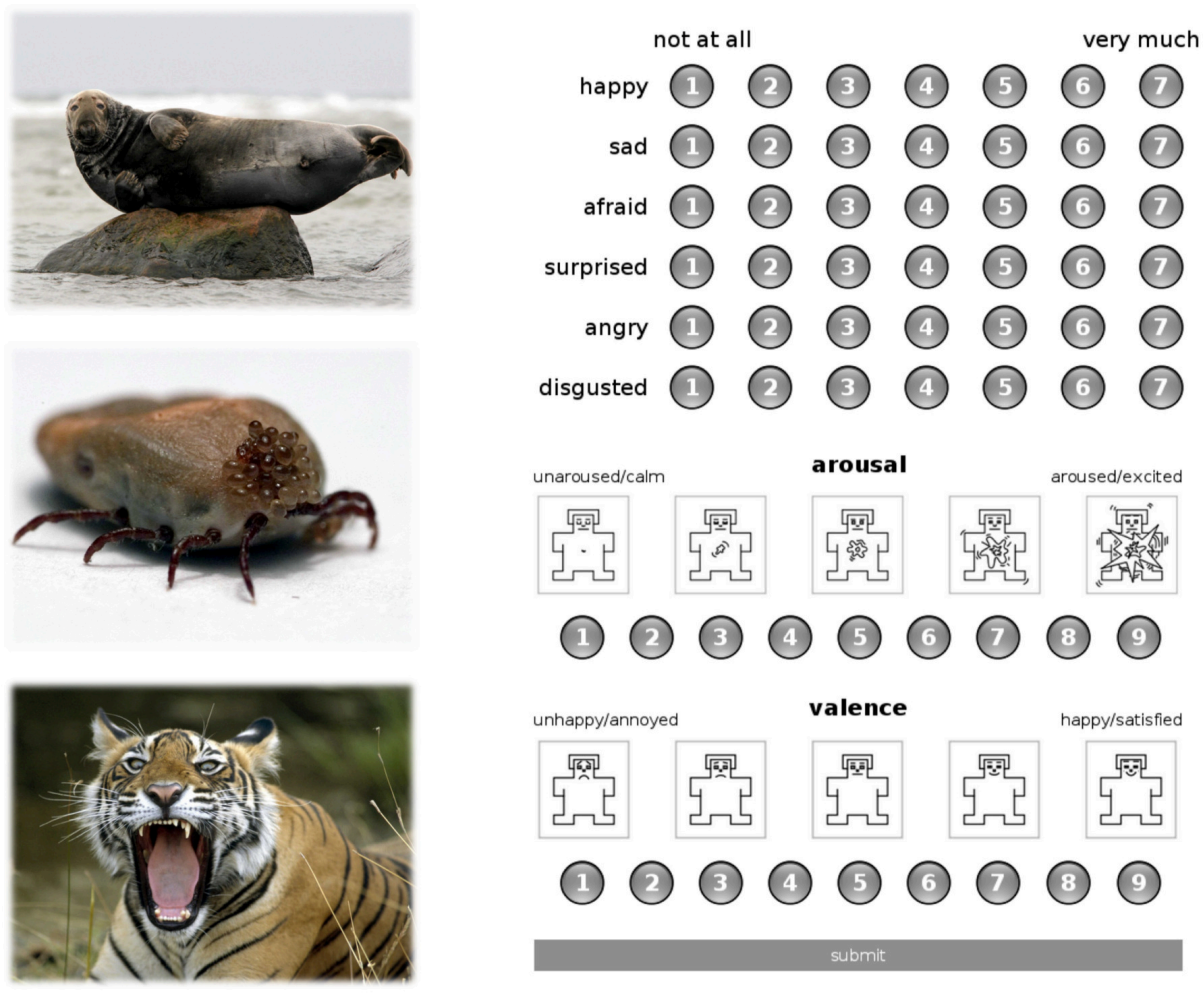
**Exemplary pictorial stimuli from the content category of animals (left panel) and the assessment platform with discrete and dimensional rating scales (right panel)**.

The study protocol consisted of two study sessions (encoding and recognition). The procedure was conducted in English, as the participants (exchange students from various European countries, recruited at the University of Warsaw) were proficient speakers of English. During the encoding session subgroup of 40 subjects (mean age = 22.4; *SD* = 3.6) from larger cohort study of Riegel et al. (2016b) without history of any neurological illness or treatment with psychoactive drugs were asked to individually rate images through a platform available on a local server. The procedure started with filling the informed consent forms and reading instructions displayed on the computer screen, with an average distance of 60 cm from the eyes. Subsequently, each participant was exposed to a consecutive presentation of one of the three picture sets, which included 170 images chosen pseudo-randomly from all the content categories and presented under the following constraints: no more than two pictures from each affective valence class (positive, neutral, negative) and no more than three pictures from each content category appeared consecutively. Participants were asked to use six independent 7-point Likert scales in order to indicate the intensity of happiness, anger, fear, sadness, disgust, and surprise (1 indicating *not at all*, and 7 indicating *a great amount*) elicited by each presented image, which enabled them to describe each image with multiple labels (Figure 1). Additionally, the pictures were rated on two affective dimensions using the Self-Assessment Manikin (SAM; Lang, 1980), as also presented in Figure 1. The scale of emotional valence was used to estimate the extent of positive or negative reaction evoked by a given picture, ranging from 1 to 9 (1 for very negative emotions and 9 for very positive emotions). On the scale of arousal, subjects estimated to what extent a particular picture makes them feel unaroused or aroused, ranging from 1 to 9 (1 for unaroused/relaxed and 9 for very much aroused, for instance agitated or excited). Single images were presented in a full-screen view for 2 s. Each presentation was followed by an exposure of the rating scales on a new screen together with a smaller picture presented in the upper part of the screen. Completing the task with no time constraints took ~45–60 min. Importantly, the participants were not informed that they would be invited to take part in the follow-up study and perform memory tests in an fMRI experiment.

Six months after the encoding session, the subjects were contacted via email and asked to participate in a follow-up fMRI study which took part in Warsaw in the period of the two following weeks with flexible choice of a days and hours. Subjects who were available at this time were screened with a standard magnetic resonance (MR) form. Seventeen subjects (10 women) were eligible to take part in the recognition session (mean age = 24.21; *SD* = 4.05). Data from 3 subjects were not eligible to analyses due to very low recognition rate (below 10%).

During the recognition test, subjects were presented with 170 old and 170 new pictures, and had to indicate whether they had seen these pictures before by pressing an appropriate button indicating OLD or NEW on a response pad after the offset of each stimulus. The fMRI experimental procedure lasted for about 34 min and was divided into two 17-min parts during which 170 stimuli were shown. Each stimulus was presented for 2 s, inter-stimulus interval varied pseudo-randomly from 5 to 7 s. The stimuli were presented pseudo-randomly under the constraints applied to the preceding encoding session and additionally no more than three pictures from each study phase (OLD/NEW) appeared consecutively. All the participants were given a financial reward of 100 PLN (~EUR 24). The local Research Ethics Committee in Warsaw at Faculty of Psychology, University of Warsaw, approved the experimental protocol of the study.

### Neuroimaging data acquisition

MRI data acquisition took place at the Laboratory of Brain Imaging, Neurobiology Center, Nencki Institute of Experimental Biology, with a 3-Tesla MR scanner (Siemens Magnetom Trio TIM, Erlangen, Germany) equipped with 32-channel phased array head coil. Functional data were acquired using a T2^*^-weighted gradient echo planar imaging (EPI) sequence with the following parameters: time repetition = 2000 ms, time echo = 25 ms, flip angle = 90⋅, in-plane resolution = 64 × 64 voxels, field of view = 224 mm, and 39 axial slices with 3.5 mm slice thickness and no gap between slices. Detailed anatomical data of the brain were acquired with a T1-weighted (T1w) sequence (time repetition = 2530 ms, time echo = 3.32 ms).

### MR data analysis

Statistical Parametric Mapping (SPM12, Wellcome Trust Center for Neuroimaging, London, UK) software running on MATLAB R2013b (The Math-Works Inc. Natick, MA, USA) was used for data pre-processing and statistical analyses. First, functional images were motion-corrected. Then, structural (T1w) images from single subjects were co-registered to the mean functional image. High-dimensional Diffeomorphic Anatomical Registration using Exponentiated Lie algebra (DARTEL) was used to create a study-specific template and flow fields based on segmented tissue from T1w images (Ashburner, 2007; Marchewka et al., 2014b). The functional images were normalized using compositions of flow fields and study-specific template to a 1.5 mm isotropic voxel size. Finally, the normalized functional images were smoothed with an 8 mm isotropic Gaussian kernel. In the first level of statistical analysis, all experimental conditions and head movement parameters were entered into a design matrix. The experimental conditions were based on behavioral responses from the memory test and included: correct recognition of OLD stimuli (remembered), incorrect recognition of OLD stimuli (forgotten), correct recognition of NEW stimuli (correct rejection), incorrect recognition of NEW stimuli (false recognition). The activity difference between remembered and forgotten items during retrieval is known as retrieval success and it was treated as a reference for the analysis of brain activity (Weis et al., 2004; Prince et al., 2005). The data was modeled for each fMRI run using the canonical hemodynamic response function co-involved with the experimental conditions.

Additionally parametric modulation analyses were computed in SPM12 software. For each subject 7 separate 1st level models were constructed. Each of the models had four conditions: correct recognition (remembered), incorrect recognition (forgotten), correct rejection, and false recognition. In each of the models, ratings of all stimuli assessed by particular subject on one of the seven scales (valence, arousal, happiness, surprise, sadness, fear, anger, or disgust) were used as parameters paired with stimuli in each or all four conditions.

To test whether the intensity of a particular emotion or emotional dimension affected brain activity during pictures recognition, t-contrasts were constructed as follows. In each of the 1st level models, brain activity correlating with parameter values in remembered stimuli was contrasted with brain activity correlating with the parameter values in forgotten stimuli. Such contrasts allow brain regions to be found where correlation of BOLD activity with the intensity of a particular emotion/dimension in remembered stimuli is higher than the same correlation in forgotten stimuli, thus leading to identification of regions involved in emotion-dependent memory. Finally, in the 2nd level analysis one sample *t*-tests were used to examine the significance of the obtained contrasts.

The correction for multiple comparisons was performed using the Monte Carlo simulation (3dClustSim, AFNI, http://afni.nimh.nih.gov). Only clusters with a minimum of 30 contiguous voxels and *p*_voxel_ < 0.001 were considered as significant (*p* < 0.05).

## Results

### Behavioral results

#### Emotional dimensions of correct vs. false recognition

**Figure 2:**
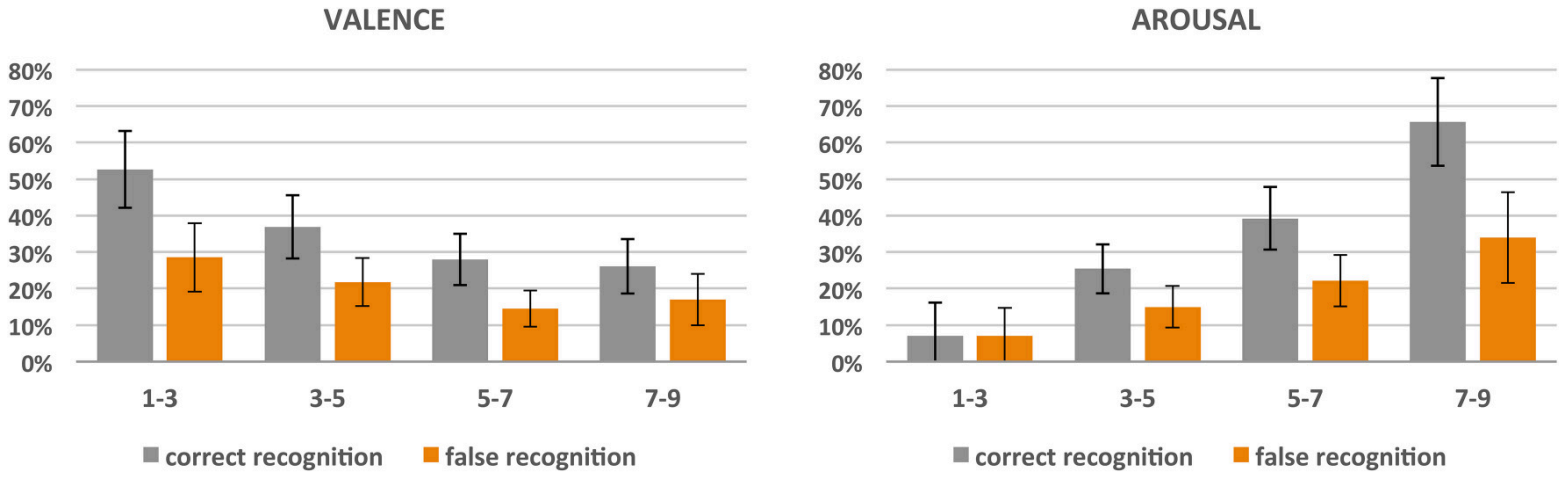
**Correct and false recognition rate (%) of pictures divided into 4 classes with respect to the obtained scores of valence and arousal**.

In order to investigate the recognition accuracy on the behavioral level, we divided the stimuli into 4 classes of valence and 4 classes of arousal based on the mean subjective ratings given on these scales. Class 1 included scores ranging from 1 to 3 (i.e., negative or low-arousing), class 2 included scores ranging from 3 to 5, class 3—scores from 5 to 7, and class 4—scores from 7 to 9 (i.e., positive or high-arousing). A repeated measure ANOVA with 4 classes of ratings and memory on two levels (correct recognition and false recognition) was used separately for valence and arousal dimensions as dependent variables. Significant effects were further examined with pairwise comparisons (see Figure 2).

In the case of valence, significant effects of both valence classes [*F*_(3, 11)_ = 12.16; *p* < 0.001; $\eta_{p}^{2}$ = 0.77) and recognition accuracy [*F*_(1, 13)_ = 63.42; *p* < 0.001; $\eta_{p}^{2}$ = 0.83] were revealed, and there was an interaction between the two [*F*_(3, 11)_ = 4.53, *p* < 0.05; $\eta_{p}^{2}$ = 0.55]. In general, the more negative the pictures, the more correctly they were remembered (class 1 was more correctly recognized than all others, *p* < 0.001; class 2 was more correctly recognized than class 3 (*p* < 0.001) and 4 (*p* < 0.005); there was no significant difference between classes 3 and 4). Nevertheless, negative pictures provoked more false recognitions than neutral and positive ones (class 1 had higher amount of false recognition than class 3 (*p* > 0.001) and 4 (*p* > 0.01); also class 2 had higher amount of false recognition than class 3 (*p* > 0.005) and 4 (*p* > 0.05).

Similar effects were found for arousal dimension: both arousal classes [*F*_(3, 11)_ = 14.68; *p* < 0.001; $\eta_{p}^{2}$ = 0.80] and recognition accuracy [*F*_(1, 13)_ = 31.26, *p* < 0.001; $\eta_{p}^{2}$ = 0.71] effects were revealed, and there was an interaction between the two [*F*_(3, 11)_ = 8.34; *p* > 0.005; $\eta_{p}^{2}$ = 0.69]. The more arousing the pictures, the more correctly they were recognized and the more false recognitions they produced. With respect to correctness of recognition, for class 4 (i.e., highly arousing) it was higher than for the others (all the differences were significant, *p* < 0.001), class 3 was recognized more correctly than classes 2 and 1 (for both *p* < 0.001), while class 2 had a higher recognition correctness rate than class 1 (*p* < 0.05). As far as false recognitions are concerned, highly arousing stimuli (class 4) had a higher rate than all the other stimuli (*p* < 0.05 for class 3 and *p* < 0.01 for classes 2 and 1), class 3 had a higher rate than classes 2 (*p* < 0.005) and 1 (*p* < 0.05), while there was no difference for low-arousing classes 2 and 1.

Importantly, the correct recognition rate (mean 33%, *SD* = 15%) was significantly higher than the rate of false recognition (mean 19%, *SD* = 12%), irrespective of subjective ratings of valence and arousal. The correct recognition rate ranged from 13 to 60% of pictures.

#### Basic emotions and emotional dimensions of remembered vs. forgotten images

**Figure 3:**
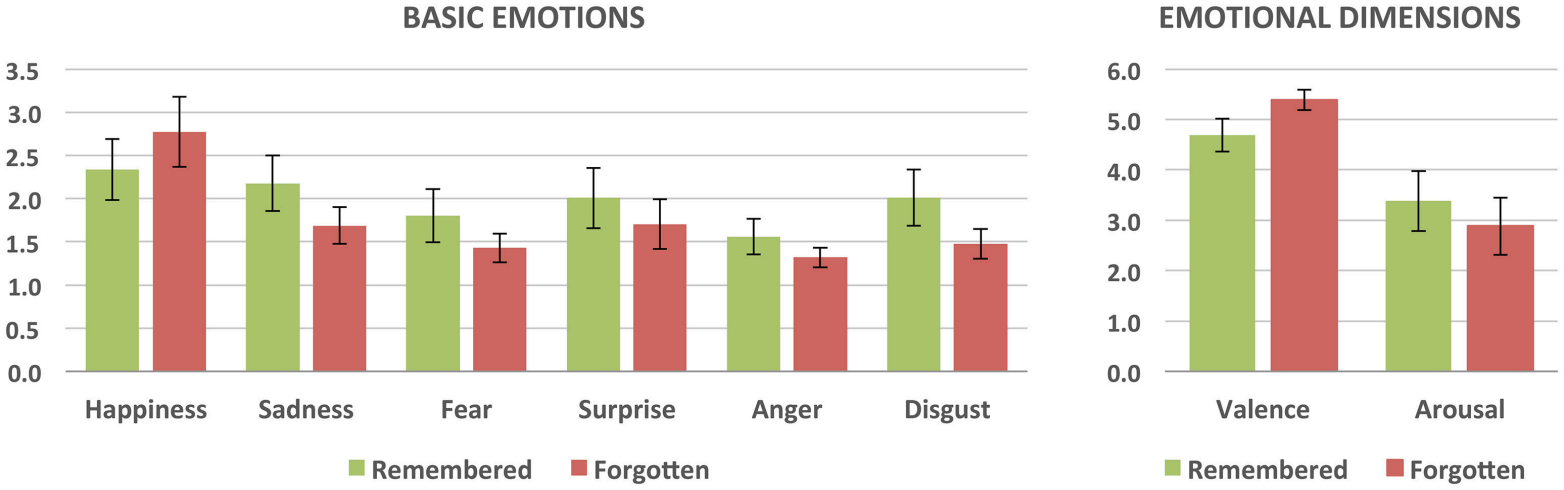
**Subjective ratings of the intensity of basic emotions and emotional dimensions for remembered (correctly recognized) and forgotten (incorrectly recognized) stimuli**. All the differences are statistically significant.

We also investigated whether the retrieval success differed as a function of basic emotion and emotional dimension intensities (see Figure 3). Paired *t*-tests for correctly recognized vs. forgotten images were performed for the mean intensities of each of the basic emotions (happiness, surprise, sadness, fear, anger, and disgust) as well as valence and arousal. The tests revealed that recognized pictures differed from the forgotten ones in both the intensity of basic emotions and emotional dimensions. Correctly recognized pictures had a lower intensity of happiness [*t*_(13)_ = −4.26; *p* < 0.001; Cohen's *d* = −1.12] and higher intensity ratings of surprise [*t*_(13)_ = 2.66; *p* < 0.05; Cohen's *d* = 0.74], sadness [*t*_(13)_ = 5.29; *p* < 0.001; Cohen's *d* = 1.79], fear [*t*_(13)_ = 3.58; *p* < 0.005; Cohen's *d* = 1.37], anger [*t*_(13)_ = 3.65; *p* < 0.005; Cohen's *d* = 1.41], and disgust [*t*_(13)_ = 4.09; *p* < 0.001; Cohen's *d* = 1.34] than forgotten images. They were also rated as more unpleasant [*t*_(13)_ = −5.55; *p* < 0.001; Cohen's *d* = −1.73] and more arousing [*t*_(13)_ = 2.91; *p* < 0.05; Cohen's *d* = 0.78].

### fMRI results

**Figure 4:**
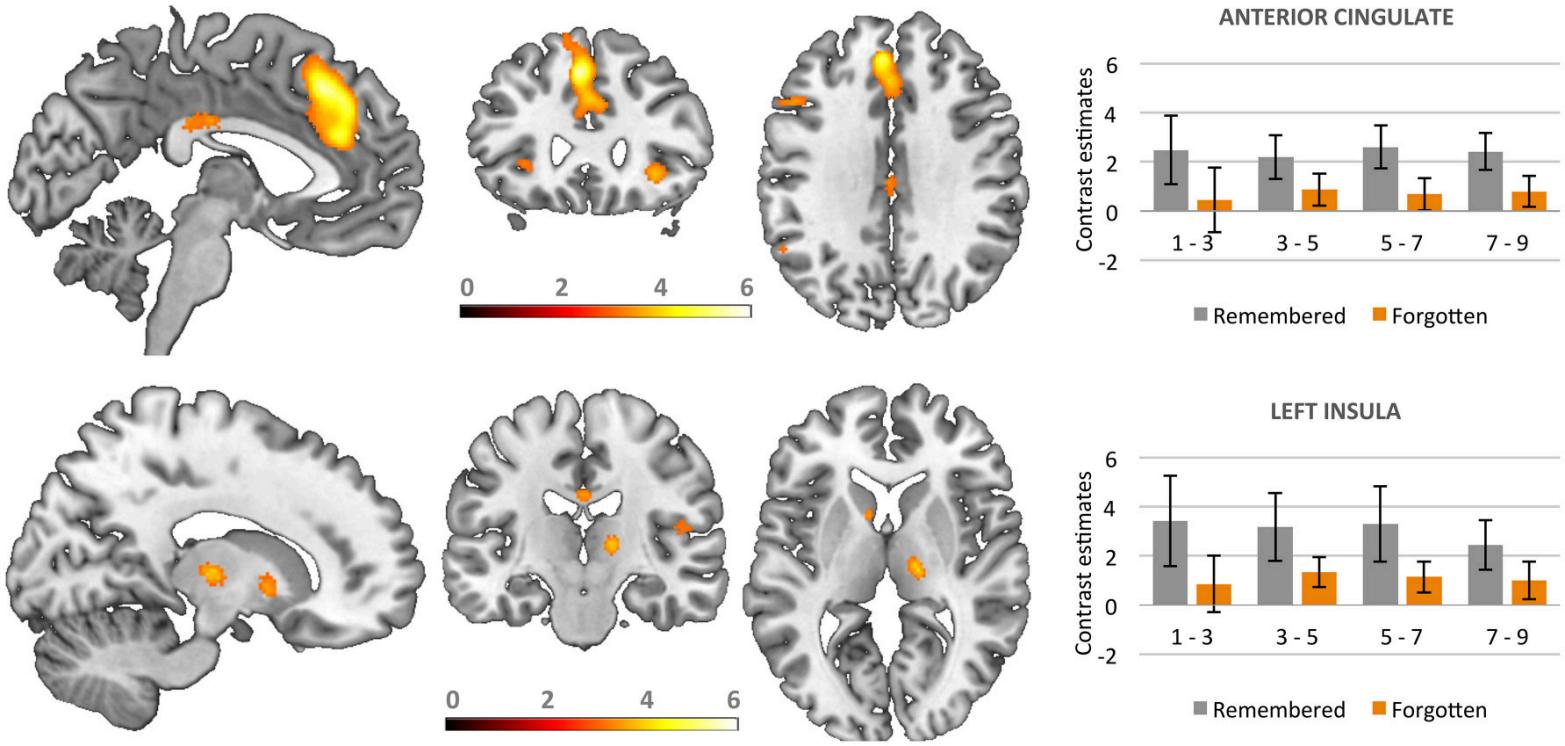
**Activations in brain areas associated with the successful recognition of emotional and neutral stimuli**. Bars represent contrast estimates for valence classes.

Whole brain analyses aiming at direct comparison of correctly recognized stimuli with forgotten ones were computed. We found significant activation for recognized vs. forgotten images in several cortical areas, including the anterior cingulate cortex (ACC) extending to superior medial frontal, bilateral anterior insula, and inferior frontal gyri as well as the left inferior parietal cortex. Increased activity for recognized as compared to forgotten pictures was also found sub-cortically in the bilateral caudate and right thalamus (see Table 1 and Figure 4).

**Table 1:** **Brain areas associated with the long-term memory retrieval success**.

**Brain region**	***T***	**MNI**			**Cluster size**
		***x***	***y***	***z***	
L superior medial frontal gyrus	5.91	−4	30	44	2311
L superior medial frontal gyrus	5.55	−6	38	39	
L anterior cingulate	4.92	−3	36	24	
L inferior frontal gyrus (orbital part)	4.94	−42	20	−10	1037
L_insula	4.46	−33	21	−3	
L_insula	4.09	−27	24	−8	
L middle frontal gyrus (orbital part)	4.44	−51	50	−6	140
R thalamus	4.31	15	−18	6	145
R_insula	4.26	32	26	−6	593
R inferior frontal gyrus (orbital part)	4.01	48	21	−9	
R inferior frontal gyrus (orbital part)	3.22	58	20	−3	
L caudate	4.01	−9	9	0	147
L pallidum	3.38	−15	3	−6	
R caudate	3.96	12	9	−2	123
L middle cingulate	3.85	−2	−20	32	236
L inferior parietal lobule	3.77	−34	−57	42	241
L inferior parietal lobule	3.47	−42	−57	50	
L middle temporal gyrus	3.71	−64	−27	−12	75
L middle frontal gyrus	3.65	−46	20	39	278
L middle frontal gyrus	3.54	−42	18	51	
L inferior frontal gyrus (operculum)	3.47	−54	20	34	
L inferior parietal lobule	3.58	−52	−56	36	85
L angular gyrus	3.18	−45	−58	40	
R rolandic operculum, insula	3.48	42	−21	18	125
R rolandic operculum	3.31	56	−14	12	

Analyses are based on all classes of valence ranging from negative to positive. All of the listed brain regions were cluster corrected and met the significance threshold of p < 0.05. The x, y, z coordinates refer to the Montreal Neurological Institute (MNI) space. L is left; R is right. Cluster size is the number of voxels activated in the regional cluster. Only the main peaks of activation within each cluster and their corresponding brain structures are reported.

**Figure 5:**
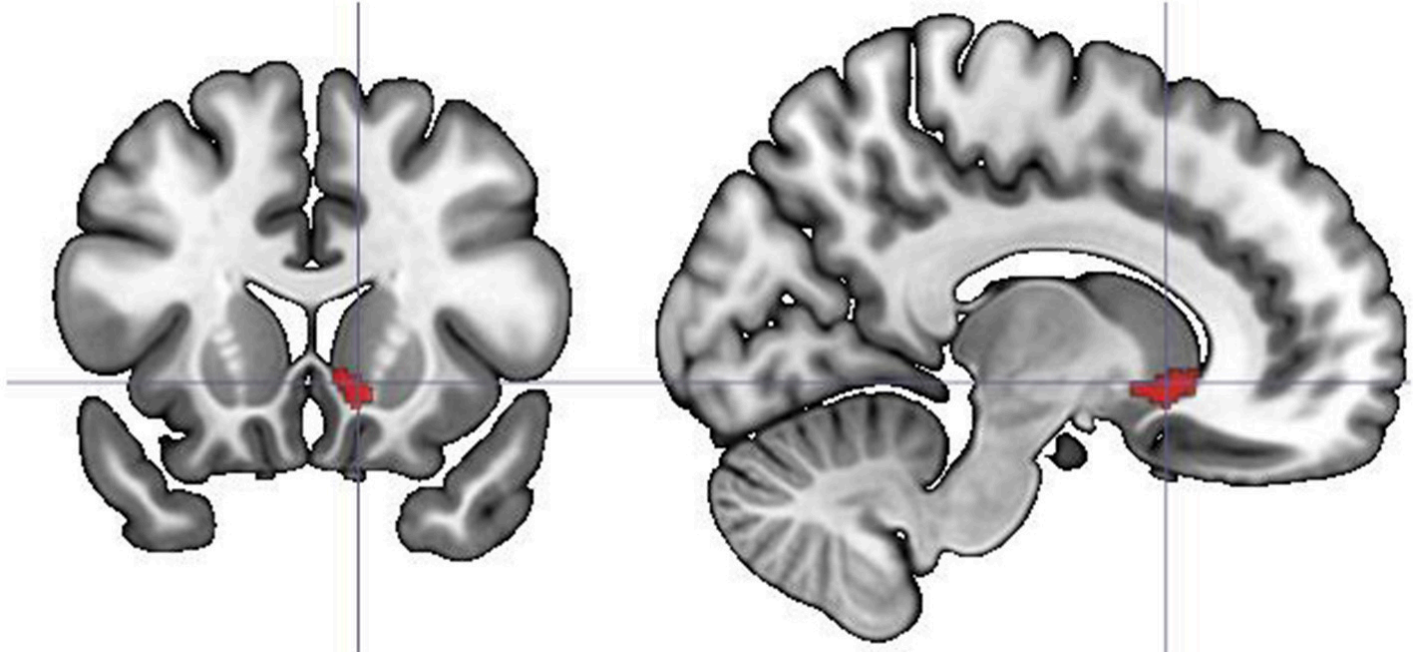
**Parametric modulation of the brain activation during long-term memory recognition by self-reported arousal ratings**. Figure presents the contrast of correlations of BOLD signal with arousal ratings between remembered and forgotten stimuli. No significant effect on analogous contrast was found for other ratings collected in line with dimensional and discrete models of affect.

Additional analyses based on parametric modulation were computed for each of the subjective ratings collected in line with dimensional and discrete models of emotion. Arousal was found to be the only subjective rating significantly modulating brain activation. Increases in subjective ratings of arousal of experimental stimuli led to increased recruitment of the right caudate (*x y z*: 6, 12, 5; *T* = 4.95, 192 voxels) and left anterior cingulate gyrus (*x y z*: −18, 24, 32; *T* = 6.06, 117 voxels) during correct recognition (see Figure 5).

## Discussion

The present study was aimed at extending the previous literature reporting long-term recognition memory effects for emotional stimuli (Bradley et al., 1992; Dolcos et al., 2005; Weymar et al., 2011; Harand et al., 2012). We were particularly interested in exploring the modulatory effect of emotions on long-term recognition memory by using normative behavioral ratings collected in line with dimensional and discrete models of affect. For this reason we developed a computerized rating procedure and conducted the memory test after a period of 6 months.

At the behavioral level, separate analyses examining the effect of emotion on memory performance according to both models of affect were computed. These analyses revealed that long-term recognition memory was significantly modulated by emotion conceptualized according to both theoretical models. Specifically, we found a significant effect of valence and arousal on recognition accuracy. Recognition accuracy was the highest for negative arousing stimuli, in line with previous studies where various types of material and methodological approaches were used (Bradley et al., 1992; Cahill and McGaugh, 1995; Ochsner, 2000; Kensinger et al., 2003; Dolcos et al., 2005). With respect to the ratings collected in line with discrete models of affect, we found emotional enhancement of memory effect only for negative emotions (sadness, fear, surprise, anger, and disgust). This effect was most pronounced in the case of disgust. Recent studies have already shown that stimuli eliciting disgust are able to capture and hold our attention much more effectively than fear-evoking images (Van Hooff et al., 2013; van Hooff et al., 2014) and are better remembered after a short retention period (Croucher et al., 2011; Marchewka et al., 2016). However, it is still debated whether disgust, since it is evidently a basic sensory affect (Rozin and Fallon, 1987) as well as a socially constructed moral emotion (Haidt, 2003), should be classified as a basic emotion (for discussion see Panksepp, 2007; Toronchuk and Ellis, 2007; Panksepp and Watt, 2011).

In the case of positive emotions (happiness) we found an opposite effect. A more positive rating of the stimuli corresponded with worse recognition success being observed. This result is in line with findings concerning the dimension of valence, reported also in the previous studies (Kensinger and Corkin, 2003; Kensinger et al., 2006, 2007). According to the proposed explanation, negative information may be encoded more automatically than positive information (Kern et al., 2005; Talmi et al., 2007), and negative items tend to be encoded with more perceptual processing than positive information (Mickley and Kensinger, 2008; Mickley Steinmetz and Kensinger, 2009). However, it is important to emphasize that the current sample was limited to young adults and observed behavioral effects could be different in older population. For example a large-scale meta-analysis showed that older adults demonstrate a significant information processing bias toward positive vs. negative information, whereas in younger adults an opposite effect was revealed (Reed et al., 2014). Nevertheless, age related effects on long-term memory have not been yet tested with long retention periods, such as the one in the current study.

At the neuronal level, using basic contrast-based fMRI analyses we found a number of brain areas associated with long-term recognition memory of emotional information, both cortical and subcortical. Cortical regions we found were previously indicated in the studies of various kinds of memory retrieval and recognition (Stock et al., 2009; Sitaram et al., 2014). Interestingly, some of these regions, i.e., the bilateral anterior insula and anterior cingulate cortex, are the core regions of the so-called salience network (Seeley et al., 2007). More and more evidence suggests that they play a critical role in mediating the interplay between brain areas involved in emotion perception and executive control (Menon and Uddin, 2010). A recent fMRI study on the influence of emotions on working memory found that the salience network plays a critical role in mediating interaction between emotion perception and executive control (Luo et al., 2014). Anatomically, core salience network regions (AI and ACC) have strong reciprocal connections with an emotional processing network including the amygdala and ventral sensory pathways (Krämer et al., 2014). It seems, based on the presented findings, that the regions of the salience network are also involved in successful recognition of stimuli after long retention periods.

In contrast to the previous study of Dolcos et al. (2005), we failed to observe any significant involvement of the hippocampus and amygdala in correct recognition of emotional items. This might be related to the fact that we computed fMRI analyses on the whole brain, whereas the results of the mentioned study were based on ROI analyses. Also, that study included only female participants, which might have influenced the pattern of brain activity. However, it seems that the involvement of the hippocampus and amygdala in long-term recognition memory may not be critical. It was first clinically demonstrated in HM patients that despite damage to the hippocampus resulting in profound amnesia, older memories, or information could still be consciously recollected and recognized. This fact led to a proposal that after a longer retention period information initially processed in the hippocampus is stored in a distributed cortical network. The ACC has recently been indicated in this process. Harand et al. (2012) found that hippocampal activity decreased over time for initially episodic, later semantic memories. For both types of memories, neocortical activations were observed with both delay periods (3 days and 3 months), specifically in the ventromedial prefrontal and anterior cingulate cortices. The authors argued that these cortical activations might reflect a gradual reorganization of memory traces within neural networks. In line with this result, studies on rodents have demonstrated that the ACC is necessary for recalling behaviors learned at least 1 month ago, but not for the same behaviors learned the preceding day (Weible et al., 2012).

The increased activity for correctly recognized compared to forgotten stimuli was also observed in the striatum, specifically in bilateral caudate and left pallidum as well as in the right thalamus. A recent study which explored the influence of genetic polymorphisms on long-term memory found that older people with the presence of an A1 allele of the DRD2/ANKK1-TaqIA polymorphism had worse memory performance and a weaker activation in left caudate nucleus (Persson et al., 2015). Further, a positive relationship between caudate activation and long-term memory updating performance among non-A1 carriers indicated that caudate activation was behaviorally relevant. Since this polymorphism is related to a reduced density of striatal dopamine D2 receptors (Jönsson et al., 1999), these results point to the critical role of dopamine in regulating long-term memory. In line with this hypothesis, emerging evidence from human and animal studies suggests that suboptimal dopamine modulation may be associated with increased forgetting of episodic information (Papenberg et al., 2013). What is more, the activation of the right thalamus found in the present study is consistent with neuropsychological findings in patients showing a double dissociation in material-specific long-term memory i.e., right thalamus lesion specifically impairs visual memory, while lesions of the left thalamus impairs verbal memory (Edelstyn et al., 2012).

Additional fMRI parametric analyses identified regions that were uniquely and increasingly recruited with a corresponding increase in subjective ratings of arousal. This modulation of brain activity by arousal was observed in the right caudate and the left anterior cingulate gyrus. Arousal was the only subjective rating significantly modulating brain activation. It seems therefore that these structures are not only significantly involved in long-term recognition memory but also in perceiving arousal *per-se*. Previous studies which showed brain correlates of arousal did not employ recognition memory paradigms but required participants to experience emotions or evaluate affective stimuli (Colibazzi et al., 2010; Viinikainen et al., 2010), thus it is difficult to directly compare them with the results from the present study. Nevertheless, they revealed the existence of distinctive brain networks managing emotions in line with the dimensional model of affect including the caudate and cingulate cortex. The activation patterns found in the present study probably reflect more complex brain networks serving both long-term recognition of emotional stimuli and experiencing of the feelings elicited by the material. Enhanced memory formation for unpleasant arousing photographs seems to be initiated already at stimulus encoding. In fact, increased stimulus processing is supposed to lead to the formation of inter-item associations and enhanced memory consolidation (Craik and Lockhart, 1972; Craik and Tulving, 1975; Cowan, 1995). Moreover, the exposure to unpleasant arousing stimuli is associated with increased retrieval of negative memories and unpleasant post-event recollection (Cuthbert et al., 2003). Superior memory formation for unpleasant arousing events seems to be adaptive, but it may also result in behavioral withdrawal and reinforce negative beliefs. These processes may impair our psychological functioning and, in extreme cases, assist in the development of mental disorders that may be difficult to alleviate because of poorer positive memory formation.

## Limitations of the study and conclusions

A strong limitation of the current study is the fact that we were unable to find significant differences in brain activations underlying long-term recognition memory in respect of subjective ratings collected in both the dimensional and discrete models of affect. These differences were visible only at the level of behavioral analyses.

We hypothesize that this lack of significant results in fMRI analyses could be a consequence of the small number of trials within each experimental condition and the relatively limited number of subjects in the recognition session with analyzable data. At the same time on the behavioral level we obtained moderate to high effect sizes. Therefore, we suggest that further experiments increase both the sample size and the number of stimuli. We also think that further studies in older adults should be conducted in order to understand the mechanisms of long-term recognition memory in humans in more details.

The most important conclusion that could be drawn from the current study is that only subjective ratings of arousal are directly associated with brain activation during long-term memory recognition. This findings may be interpreted against theories of basic emotions postulating that a discrete and independent neural system subserves every emotion (Posner et al., 2005; see also Clark-Polner et al., 2016).

In addition, the obtained results also support the hypothesis that the memorized material is firstly processed in the hippocampus and amygdala, yet over time it becomes stored in a distributed cortical network including the core salience network and basal ganglia.

## Author contributions

AM was responsible for the study concept and design contributed to the acquisition of the data, performed the data analysis and wrote the article. MW performed the data analysis. AMo acquisition of the data. MR contributed to the writing of the article. JM study concept and design contributed to the writing of the article. KJ performed the data analysis and wrote the article. All authors critically reviewed content and approved final version for publication.

### Conflict of interest statement

The authors declare that the research was conducted in the absence of any commercial or financial relationships that could be construed as a potential conflict of interest.

## References

[B1] Ashburner J. (2007). A fast diffeomorphic image registration algorithm. Neuroimage 38, 95–113. 10.1016/j.neuroimage.2007.07.007 17761438

[B2] Bradley M. M. Greenwald M. K. Petry M. C. Lang P. J. (1992). Remembering pictures: pleasure and arousal in memory. J. Exp. Psychol. Learn. Mem. Cogn. 18, 379–390. 10.1037/0278-7393.18.2.379 1532823

[B3] Briesemeister B. B. Kuchinke L. Jacobs A. M. (2011). Discrete emotion norms for nouns: Berlin affective word list (DENN-BAWL). Behav. Res. Methods 43, 441–448. 10.3758/s13428-011-0059-y 21416309

[B4] Brosch T. Scherer K. R. Grandjean D. Sander D. (2013). The impact of emotion on perception, attention, memory, and decision-making. Swiss Med. Wkly. 143, 1–10. 10.4414/smw.2013.13786 23740562

[B5] Cahill L. McGaugh J. L. (1995). A novel demonstration of enhanced memory associated with emotional arousal. Conscious. Cogn. 4, 410–421. 10.1006/ccog.1995.1048 8750416

[B6] Chapman H. A. Johannes K. Poppenk J. L. Moscovitch M. Anderson A. K. (2012). Evidence for the differential salience of disgust and fear in episodic memory. J. Exp. Psychol. Gen. 142, 1100–1112. 10.1037/a0030503 23067064

[B7] Charash M. McKay D. (2002). Attention bias for disgust. J. Anxiety Disord. 16, 529–541. 10.1016/S0887-6185(02)00171-8 12396210

[B8] Clark-Polner E. Johnson T. D. Barrett L. F. (2016). Multivoxel pattern analysis does not provide evidence to support the existence of basic emotions. Cereb. Cortex. [Epub ahead of print]. 10.1093/cercor/bhw028 26931530 PMC6093086

[B9] Colibazzi T. Posner J. Wang Z. Gorman D. Gerber A. Yu S.. (2010). Neural systems subserving valence and arousal during the experience of induced emotions. Emotion 10, 377–389. 10.1037/a0018484 20515226

[B10] Craik F. I. M. Lockhart R. S. (1972). Levels of processing: a framework for memory research. J. Verb. Learn. Verb. Behav. 11, 671–684.

[B11] Craik F. I. M. Tulving E. (1975). Depth of processing and the retention of words in episodic memory. J. Exp. Psychol. Gen. 104, 268–294.

[B12] Croucher C. J. Calder A. J. Ramponi C. Barnard P. J. Murphy F. C. (2011). Disgust enhances the recollection of negative emotional images. PLoS ONE 6:e26571. 10.1371/journal.pone.0026571 22110588 PMC3217922

[B13] Cowan N. (1995). Attention and Memory: An Integrated Framework. Oxford Psychology Series (No. 26), (Paperback edition: 1997). New York, NY: Oxford University Press.

[B14] Cuthbert B. N. Lang P. J. Strauss C. Drobes D. Patrick C. J. Bradley M. M. (2003). The psychophysiology of anxiety disorder: fear memory imagery. Psychophysiology 40, 407–422. 10.1111/1469-8986.00043 12946114

[B15] Dolcos F. Denkova E. (2014). Current emotion research in cognitive neuroscience: linking enhancing and impairing effects of emotion on cognition. Emot. Rev. 6, 362–375. 10.1177/1754073914536449

[B16] Dolcos F. LaBar K. S. Cabeza R. (2004). Interaction between the amygdala and the medial temporal lobe memory system predicts better memory for emotional events. Neuron 42, 855–863. 10.1016/S0896-6273(04)00289-2 15182723

[B17] Dolcos F. LaBar K. S. Cabeza R. (2005). Remembering one year later: role of the amygdala and the medial temporal lobe memory system in retrieving emotional memories. Proc. Natl. Acad. Sci. U.S.A. 102, 2626–2631. 10.1073/pnas.0409848102 15703295 PMC548968

[B18] Dolcos F. Wang L. Mather M. (2014). Current research and emerging directions in emotion-cognition interactions. Front. Integr. Neurosci. 8:83. 10.3389/fnint.2014.00083 25426034 PMC4227476

[B19] Edelstyn N. M. J. Mayes A. R. Denby C. Ellis S. J. (2012). Impairment in material-specific long-term memory following unilateral mediodorsal thalamic damage and presumed partial disconnection of the mammillo-thalamic tract. J. Neuropsychol. 6, 119–140. 10.1111/j.1748-6653.2011.02019.x 22257705

[B20] Ekman P. (1992). An argument for basic emotions. Cogn. Emot. 6, 169–200. 10.1080/02699939208411068

[B21] Haidt J. (2003). The moral emotions, in Handbook of Affective Sciences, eds Davidson R. J. Scherer K. R. Goldsmith H. H. (New York, NY: Oxford University Press), 852–870.

[B22] Harand C. Bertran F. La Joie R. Landeau B. Mézenge F. Desgranges B.. (2012). The hippocampus remains activated over the long term for the retrieval of truly episodic memories. PLoS ONE 7:e43495. 10.1371/journal.pone.0043495 22937055 PMC3427359

[B23] Holland A. C. Kensinger E. A. (2013). Emotion in episodic memory. the effects of emotional content, emotional state, and motivational goals, in The Cambridge Handbook of Affective Neuroscience, eds Armony J. Vuilleumier P. (New York, NY: Cambridge University Press), 465–488.

[B24] Jönsson E. G. Nöthen M. M. Grünhage F. Farde L. Nakashima Y. Propping P.. (1999). Polymorphisms in the dopamine D2 receptor gene and their relationships to striatal dopamine receptor density of healthy volunteers. Mol. Psychiatry 4, 290–296. 10.1038/sj.mp.4000532 10395223

[B25] Kalpouzos G. Fischer H. Rieckmann A. MacDonald S. W. S. Bäckman L. (2012). Impact of negative emotion on the neural correlates of long-term recognition in younger and older adults. Front. Integr. Neurosci. 6:74. 10.3389/fnint.2012.00074 23049503 PMC3445868

[B26] Kensinger E. A. Clarke R. J. Corkin S. (2003). What neural correlates underlie successful encoding and retrieval? A functional magnetic resonance imaging study using a divided attention paradigm. J. Neurosci. 23, 2407–2415. 12657700 10.1523/JNEUROSCI.23-06-02407.2003 PMC6742028

[B27] Kensinger E. A. Corkin S. (2003). Memory enhancement for emotional words: are emotional words more vividly remembered than neutral words? Mem. Cogn. 31, 1169–1180. 10.3758/BF03195800 15058678

[B28] Kensinger E. A. Garoff-Eaton R. J. Schacter D. L. (2006). Memory for specific visual details can be enhanced by negative arousing content. J. Mem. Lang. 54, 99–112. 10.1016/j.jml.2005.05.005

[B29] Kensinger E. A. Garoff-Eaton R. J. Schacter D. L. (2007). How negative emotion enhances the visual specificity of a memory. J. Cogn. Neurosci. 19, 1872–1887. 10.1162/jocn.2007.19.11.1872 17958489

[B30] Kern R. P. Libkuman T. M. Otani H. Holmes K. (2005). Emotional stimuli, divided attention, and memory. Emotion 5, 408–417. 10.1037/1528-3542.5.4.408 16366745

[B31] Krämer K. Bente G. Kuzmanovic B. Barisic I. Pfeiffer U. J. Georgescu A. L. (2014). Neural correlates of emotion perception depending on culture and gaze direction. Cult. Brain 2, 27–51. 10.1007/s40167-014-0013-9

[B32] Lang P. J. (1980). Behavioral treatment and bio-behavioral assessment: computer applications, in Technology in Mental Health Care Delivery Systems, eds Sidowski J. B. Johnson J. H. Williams T. A. (Norwood, NJ: Ablex), 119–137.

[B33] Libkuman T. M. Otani H. Kern R. Viger S. G. Novak N. (2007). Multidimensional normative ratings for the international affective picture system. Behav. Res. Methods 39, 326–334. 10.3758/BF03193164 17695361

[B34] Luo Y. Qin S. Fernández G. Zhang Y. Klumpers F. Li H. (2014). Emotion perception and executive control interact in the salience network during emotionally charged working memory processing. Hum. Brain Mapp. 35, 5606–5616. 10.1002/hbm.22573 25044711 PMC6869603

[B35] Marchewka A. Kherif F. Krueger G. Grabowska A. Frackowiak R. Draganski B. (2014a). Influence of magnetic field strength and image registration strategy on voxel-based morphometry in a study of Alzheimer's disease. Hum. Brain Mapp. 35, 1865–1874. 10.1002/hbm.22297 23723177 PMC6869431

[B36] Marchewka A. Wypych M. Michalowski J. M. Sińczuk M. Wordecha M. Jednoróg K.. (2016). What is the effect of basic emotions on directed forgetting? Investigating the role of basic emotions in memory. Front. Hum. Neurosci. 10:378. 10.3389/fnhum.2016.00378 27551262 PMC4976095

[B37] Marchewka A. Żurawski Ł. Jednoróg K. Grabowska A. (2014b). The nencki affective picture system (NAPS): introduction to a novel, standardized, wide-range, high-quality, realistic picture database. Behav. Res. Methods 46, 596–610. 10.3758/s13428-013-0379-1 23996831 PMC4030128

[B38] McGaugh J. L. (2004). The amygdala modulates the consolidation of memories of emotionally arousing experiences. Annu. Rev. Neurosci. 27, 1–28. 10.1146/annurev.neuro.27.070203.144157 15217324

[B39] Menon V. Uddin L. Q. (2010). Saliency, switching, attention and control: a network model of insula function. Brain Struct. Funct. 214, 655–667. 10.1007/s00429-010-0262-0 20512370 PMC2899886

[B40] Mickley K. R. Kensinger E. A. (2008). Emotional valence influences the neural correlates associated with remembering and knowing. Cogn. Affect. Behav. Neurosci. 8, 143–152. 10.3758/CABN.8.2.143 18589505

[B41] Mickley Steinmetz K. R. Kensinger E. A. (2009). The effects of valence and arousal on the neural activity leading to subsequent memory. Psychophysiology 46, 1190–1199. 10.1111/j.1469-8986.2009.00868.x 19674398 PMC2875869

[B42] Mikels J. A. Fredrickson B. L. Larkin G. R. Lindberg C. M. Maglio S. J. Reuter-Lorenz P. A. (2005). Emotional category data on images from the International Affective Picture System. Behav. Res. Methods 37, 626–630. 10.3758/BF03192732 16629294 PMC1808555

[B43] Ochsner K. N. (2000). Are affective events richly recollected or simply familiar? The experience and process of recognizing feelings past. J. Exp. Psychol. Gen. 129, 242–261. 10.1037/0096-3445.129.2.242 10868336

[B44] Osgood C. E, Suci, G. Tannenbaum P. (1957). The Measurement of Meaning. Urbana, IL: University of Illinois.

[B45] Panksepp J. (2007). Criteria for basic emotions: is DISGUST a primary “emotion”? Cogn. Emot. 21, 1819–1828. 10.1080/02699930701334302

[B46] Panksepp J. Watt D. (2011). What is basic about basic emotions? Lasting lessons from affective neuroscience. Emot. Rev. 3, 387–396. 10.1177/1754073911410741

[B47] Papenberg G. Bäckman L. Nagel I. E. Nietfeld W. Schröder J. Bertram L.. (2013). Dopaminergic gene polymorphisms affect long-term forgetting in old age: further support for the magnification hypothesis. J. Cogn. Neurosci. 25, 571–9. 10.1162/jocn_a_00359 23363412

[B48] Persson J. Rieckmann A. Kalpouzos G. Fischer H. Bäckman L. (2015). Influences of a DRD2 polymorphism on updating of long-term memory representations and caudate BOLD activity: magnification in aging. Human Brain Mapp. 36, 1325–1334. 10.1002/hbm.22704 25486867 PMC6869631

[B49] Posner J. Russell J. A. Peterson B. S. (2005). The circumplex model of affect: an integrative approach to affective neuroscience, cognitive development, and psychopathology. Dev. Psychopathol. 17, 715–734. 10.1017/S0954579405050340 16262989 PMC2367156

[B50] Prince S. E. Daselaar S. M. Cabeza R. (2005). Neural correlates of relational memory: successful encoding and retrieval of semantic and perceptual associations. J. Neurosci. 25, 1203–1210. 10.1523/JNEUROSCI.2540-04.2005 15689557 PMC6725951

[B51] Reed A. E. Chan L. Mikels J. A. (2014). Meta-analysis of the age-related positivity effect: age differences in preferences for positive over negative information. Psychol. Aging 29, 1–15. 10.1037/a0035194 24660792

[B52] Reisenzein R. (1994). Pleasure-arousal theory and the intensity of emotions. J. Pers. Soc. Psychol. 67, 525–539. 10.1037/0022-3514.67.3.525

[B53] Riegel M. Wierzba M. Grabowska A. Jednoróg K. Marchewka A. (2016a). Effect of emotion on memory for words and their context. J. Compar. Neurol. 524, 1636–1645. 10.1002/cne.23928 26560407

[B54] Riegel M. Żurawski Ł. Wierzba M. Moslehi A. Klocek Ł. Horvat M.. (2016b). Characterization of the nencki affective picture system by discrete emotional categories (NAPS BE). Behav. Res. Methods 48, 600–612. 10.3758/s13428-015-0658-0 26205422 PMC4891391

[B55] Rozin P. Fallon A. (1987). A perspective on disgust. Psychol. Rev. 94, 23–41. 3823304

[B56] Russell J. A. (1980). A circumplex model of affect. J. Pers. Soc. Psychol. 39, 1161–1178. 10.1037/h0077714

[B57] Seeley W. W. Menon V. Schatzberg A. F. Keller J. Glover G. H. Kenna H.. (2007). Dissociable intrinsic connectivity networks for salience processing and executive control. J. Neurosci. 27, 2349–2356. 10.1523/JNEUROSCI.5587-06.2007 17329432 PMC2680293

[B58] Sitaram R. Caria A. Veit R. Gaber T. Ruiz S. Birbaumer N. (2014). Volitional control of the anterior insula in criminal psychopaths using real-time fMRI neurofeedback: a pilot study. Front. Behav. Neurosci. 8:344. 10.3389/fnbeh.2014.00344 25352793 PMC4196629

[B59] Stevenson R. A. James T. W. (2008). Affective auditory stimuli: characterization of the international affective digitized sounds (IADS) by discrete emotional categories. Behav. Res. Methods 40, 315–321. 10.3758/BRM.40.1.315 18411555

[B60] Stevenson R. A. Mikels J. A. James T. W. (2007). Characterization of the affective norms for English words by discrete emotional categories. Behav. Res. Methods 39, 1020–1024. 10.3758/BF03192999 18183921

[B61] Stock O. Röder B. Burke M. Bien S. Rösler F. (2009). Cortical activation patterns during long-term memory retrieval of visually or haptically encoded objects and locations. J. Cogn. Neurosci. 21, 58–82. 10.1162/jocn.2009.21006 18476766

[B62] Talmi D. Schimmack U. Paterson T. Moscovitch M. (2007). The role of attention and relatedness in emotionally enhanced memory. Emotion 7, 89–102. 10.1037/1528-3542.7.1.89 17352566

[B63] Toronchuk J. A. Ellis G. F. (2007). Disgust: sensory affect or primary emotional system? Cogn. Emot. 21, 1799–1818. 10.1080/0269993070129851527732652

[B64] Van Hooff J. C. Devue C. Vieweg P. E. Theeuwes J. (2013). Disgust- and not fear-evoking images hold our attention. Acta Psychol. 143, 1–6. 10.1016/j.actpsy.2013.02.001 23500108

[B65] van Hooff J. C. van Buuringen M. El M'rabet I. de Gier M. van Zalingen L. (2014). Disgust-specific modulation of early attention processes. Acta Psychol. 152, 149–157. 10.1016/j.actpsy.2014.08.009 25226546

[B66] Viinikainen M. Jääskeläinen I. P. Alexandrov Y. Balk M. H. Autti T. Sams M. (2010). Non-linear relationship between emotional valence and brain activity: evidence of separate negative and positive valence dimensions. Hum. Brain Mapp. 31, 1030–1040. 10.1002/hbm.20915 19957266 PMC6870582

[B67] Weible A. P. Rowland D. C. Monaghan C. K. Wolfgang N. T. Kentros C. G. (2012). Neural correlates of long-term object memory in the mouse anterior cingulate cortex. J. Neurosci. 32, 5598–5608. 10.1523/JNEUROSCI.5265-11.2012 22514321 PMC6703503

[B68] Weis S. Klaver P. Reul J. Elger C. E. Fernández G. (2004). Temporal and cerebellar brain regions that support both declarative memory formation and retrieval. Cereb. Cortex 14, 256–267. 10.1093/cercor/bhg125 14754866

[B69] Weymar M. Löw A. Hamm A. O. (2011). Emotional memories are resilient to time: evidence from the parietal ERP old/new effect. Hum. Brain Mapp. 32, 632–640. 10.1002/hbm.21051 21391253 PMC6870483

[B70] Ziaei M. Fischer H. (2016). Emotion and aging: the impact of emotion on attention, memory, and face recognition in late adulthood, in Neuroimaging Personality, Social Cognition, and Character, eds Absher J. R. Cloutier J. (San Diego, CA: Academic Press; Elsevier), 259–278.

